# Hydrogen Sulfide and Neurogenic Inflammation in Polymicrobial Sepsis: Involvement of Substance P and ERK-NF-κB Signaling

**DOI:** 10.1371/journal.pone.0024535

**Published:** 2011-09-12

**Authors:** Seah-Fang Ang, Shabbir M. Moochhala, Paul A. MacAry, Madhav Bhatia

**Affiliations:** 1 Immunology Program and Department of Microbiology, Center for Life Sciences, National University of Singapore, Singapore; 2 NUS Graduate School for Integrative Sciences and Engineering, National University of Singapore, Singapore; 3 Department of Pharmacology, Yong Loo Lin School of Medicine, National University of Singapore, Singapore; 4 Defense Medical and Environmental Research Institute, DSO National Laboratories, Singapore; 5 Department of Pathology, University of Otago, Christchurch, New Zealand; University of Southern California, United States of America

## Abstract

Hydrogen sulfide (H_2_S) has been shown to induce transient receptor potential vanilloid 1 (TRPV1)-mediated neurogenic inflammation in polymicrobial sepsis. However, endogenous neural factors that modulate this event and the molecular mechanism by which this occurs remain unclear. Therefore, this study tested the hypothesis that whether substance P (SP) is one important neural element that implicates in H_2_S-induced neurogenic inflammation in sepsis in a TRPV1-dependent manner, and if so, whether H_2_S regulates this response through activation of the extracellular signal-regulated kinase-nuclear factor-κB (ERK-NF-κB) pathway. Male Swiss mice were subjected to cecal ligation and puncture (CLP)-induced sepsis and treated with TRPV1 antagonist capsazepine 30 minutes before CLP. DL-propargylglycine (PAG), an inhibitor of H_2_S formation, was administrated 1 hour before or 1 hour after sepsis, whereas sodium hydrosulfide (NaHS), an H_2_S donor, was given at the same time as CLP. Capsazepine significantly attenuated H_2_S-induced SP production, inflammatory cytokines, chemokines, and adhesion molecules levels, and protected against lung and liver dysfunction in sepsis. In the absence of H_2_S, capsazepine caused no significant changes to the PAG-mediated attenuation of lung and plasma SP levels, sepsis-associated systemic inflammatory response and multiple organ dysfunction. In addition, capsazepine greatly inhibited phosphorylation of ERK_1/2_ and inhibitory κBα, concurrent with suppression of NF-κB activation even in the presence of NaHS. Furthermore, capsazepine had no effect on PAG-mediated abrogation of these levels in sepsis. Taken together, the present findings show that H_2_S regulates TRPV1-mediated neurogenic inflammation in polymicrobial sepsis through enhancement of SP production and activation of the ERK-NF-κB pathway.

## Introduction

The neuropeptide substance P (SP) is an 11 amino acid peptide encoded by the preprotachykinin-A (PPT-A) gene. It is distributed throughout the nervous system of human and animal species [1], [2]. Belonging to the tachykinin family of neurotransmitters, SP is well recognized for its numerous potent neuroimmunomodulatory actions. Its biological activities are primarily mediated through neurokinin-1 receptor. SP has been established to exert a vast range of proinflammatory effects *in vitro* and *in vivo*, influencing many immune and inflammatory disorders of the respiratory, gastrointestinal, and musculoskeletal systems [2]. SP activates inflammatory cells to produce various inflammatory molecules such as cytokines, chemokines, reactive oxygen species, and arachidonic acid derivatives that potentiate tissue inflammation, and vasoactive substances like histamine and serotonin that promote vascular leakiness and edema at the injured tissue site [1], [2]. Additionally, SP provokes lymphocyte proliferation, immunoglobulin production, leukocyte chemotaxis, and activation of proinflammatory transcription factors; all of which exacerbate tissue injury and amplify the overall inflammatory response [2]. Therefore, it is obvious that an extensive neuro-immune intersystem crosstalk exist between SP and the inflammatory response to injury.

Neurogenic inflammation encompasses a series of inflammatory responses triggered by the activation of primary sensory neurons and the subsequent release of inflammatory neuropeptides [3]. Of major importance in the development of neurogenic inflammation is the transient receptor potential vanilloid type 1 (TRPV1) receptor, a non-selective cation channel that is best characterized for its location on these neurons [4]. Besides integrating painful stimuli, TRPV1-expressing sensory nerve terminals play a dominant role in initiating neural inflammatory processes [3]. In particular, activation of TRPV1 by noxious stimuli, including hydrogen sulfide (H_2_S), leads to depolarization with consequent release of neuropeptides such as SP that participates in neurogenic inflammation [5].

In recent years, H_2_S has been recognized as the third endogenous signaling gasotransmitter, alongside carbon monoxide and nitric oxide. Cystathionine-β-synthase (CBS) in the central nervous system and cystathionine-γ-lyase (CSE) in the cardiovascular system are the key enzymes mostly responsible for the enzymatic production of H_2_S [6]. Besides acting as a vasodilator and neuromodulator [7], [8], H_2_S functions as a cardiovascular modulator and was associated with vascular consequences of endotoxic and septic shock [9]. It also contributes to local and systemic inflammation seen in experimental models of hind paw edema, acute pancreatitis, endotoxemia, and cecal ligation and puncture (CLP)-induced sepsis [6]. Importantly, our earlier work has demonstrated that H_2_S promotes TRPV1-mediated neurogenic inflammation in polymicrobial sepsis [10]. However, endogenous neural factors that modulate this event have yet to be identified.

Sepsis is characterized by systemic inflammatory response syndrome (SIRS) and multiple organ dysfunction syndrome (MODS) [11]. Sepsis develops when the initial, appropriate host response to an infection becomes amplified, and then dysregulated. Microorganisms and their products implicate in aberrant inflammatory mediator production, such as cytokines and chemokines, which in turn upregulate adhesion molecules and stimulate leukocyte recruitment [11], [12]. Activation of nuclear factor-κB (NF-κB) results in increased gene expression and biosynthesis of proinflammatory mediators in sepsis [13]. In addition, extracellular signal-regulated kinase (ERK) signaling has been shown to be important for the temporal control of NF-κB transcriptional activity and expression of NF-κB-regulated genes, as well as upstream activator of NF-κB [14], [15]. Other studies also highlighted the significance of NF-κB and ERK in sepsis [14], [15], [16], [17]. Furthermore, H_2_S was found to regulate inflammatory response in sepsis through activation of the ERK pathway [18]. However, none has investigated the detailed signaling mechanism of endogenous H_2_S in mediating neurogenic inflammation in polymicrobial sepsis in a TRPV1 relevance context. Therefore, in the present study, we hypothesized that SP is one important neural element that implicates in H_2_S-induced neurogenic inflammation in sepsis through a TRPV1 channel-dependent mechanism, and that H_2_S regulates this response through activation of the ERK-NF-κB pathway.

## Materials and Methods

### Animal model of polymicrobial sepsis

All experiments were approved by the Animal Ethics Committee of National University of Singapore and were conducted in accordance with established International Guiding Principles for Animal Research (permit number 732/08). Polymicrobial sepsis was induced by CLP as previously described [10]. Male Swiss mice (25–30 g) were anesthetized with a mixture of ketamine and medetomindine (7.5 ml/kg, i.p.) Sham mice, which served as controls, underwent the same procedure without CLP. All mice received 1 ml of saline s.c. after the surgery. Capsazepine (15 mg/kg, s.c.; Sigma-Aldrich, St. Louis, MO, USA), a TRPV1 antagonist, or vehicle (DMSO) was administrated to mice 30 minutes before surgery. The dosage of capsazepine has been described in the literature and found to be effective *in vivo* [10], [19], [20], [21], [22], [23], [24]. DL-propargylglycine (PAG; 50 mg/kg, i.p.; Sigma-Aldrich), an irreversible inhibitor of CSE, was administered either 1 hour before (“prophylactic”) or 1 hour after (“therapeutic”) surgery. Sodium hydrosulfide (NaHS; 10 mg/kg, i.p.; Sigma-Aldrich) was given to mice at the time of CLP. The doses of NaHS and PAG used are well established and have been used extensively in many animal models to study H_2_S [25], [26], [27]. Additionally, some mice received PD98059 (10 mg/kg, i.p.; Calbiochem, San Diego, CA), a potent and selective antagonist of MEK-1, which is the upstream kinase of ERK_1/2_ [28], [29]. The dose of PD98059 used is well established and has been widely used in many animal models to block the effect of ERK_1/2_ [18], [30], [31], [32], [33], [34]. PD98059 or vehicle (DMSO) was administrated to mice 1 hour before CLP. Mice were killed by a lethal dose of pentobarbitone (90 mg/kg, i.p.) 8 hours after the operation. The time point was established as described in the literature [18] and has been used in our previous work [10]. Samples of lung, liver, and blood were collected. Plasma was prepared from anticoagulated blood samples by centrifugation at 13,000 rpm for 10 minutes at 4°C. Samples were then stored at −80°C for subsequent analysis.

### Measurement of SP levels

Samples of lung and plasma were collected from the animals. Lung samples were homogenized in 1 ml of ice-cold SP assay buffer for 20 seconds (Bachem, Peninsula Laboratories, San Carlos, CA, USA). The homogenates were centrifuged (13,000 *g*, 20 minutes, 4°C) and the supernatants collected. The supernatants and plasma were adsorbed on C_18_ cartridge columns (Bachem, Peninsula Laboratories) as previously described [35]. The adsorbed peptide was eluted with 1.5 ml of 75% (v/v) acetonitrile. The samples were freeze-dried and reconstituted in the SP assay buffer (Bachem, Peninsula Laboratories). SP content in the samples was determined with an ELISA kit (Bachem, Peninsula Laboratories) according to the manufacturer's instructions. Results were then corrected for the DNA content of the tissue samples fluorometrically using Hoechst dye 33256 [36] and were expressed as nanograms per microgram of DNA.

### Cytokines, chemokines, and adhesion molecules determination

Lung and liver samples were thawed, homogenized in 20 mM phosphate buffer (pH 7.4), centrifuged (2000 *g*, 5 minutes, 4°C), and the supernatants collected. Single-analyte ELISA assays were performed for the measurement of cytokines, interleukin (IL)-1β, IL-6, tumor necrosis factor (TNF)-α; chemokines, macrophage inflammatory protein (MIP)-1α, MIP-2; and adhesion molecules, P-selectin, E-selectin, intercellular adhesion molecule (ICAM)-1, and vascular cell adhesion molecule (VCAM)-1, according to the manufacturer's instructions (R&D Systems, Minneapolis, MN, USA). The lower limits of detection for the levels of IL-1β, IL-6, TNF-α, MIP-1α, MIP-2, P-selectin, E-selectin, ICAM-1 and VCAM-1 were 15.625, 15.625, 31.25, 3.91, 15.625, 31.25, 31.25, 62.5 and 62.5 pg/ml, respectively. The ELISA results were reproducible with inter-assay variability of <9.5% and intra-assay variability of <6.5%. Results were then corrected for the DNA content of the tissue samples [36] and were expressed as picograms per microgram of DNA.

### Semiquantitative RT-PCR analysis

Total RNA was extracted with TRIzol reagent (Invitrogen Life Technologies, Carlsbad, CA, USA) according to the manufacturer's protocol. One microgram of RNA was reverse transcribed using the iScript cDNA synthesis kit (Bio-Rad, Hercules, CA, USA) at 25°C for 5 minutes, 42°C for 30 minutes, followed by 85°C for 5 minutes. The cDNA was used as a template for PCR amplification by iQ Supermix (Bio-Rad). The following primers were used: PPT-A: 5′-GCCAATGCAGAACTACGAAA-3′ and 5′-GCTTGGACAGCTCCTTCATC-3′; r18S: 5′-CCATCCAATCGGTAGTAGCG-3′ and 5′-GTAACCCGTTGAACCCCATT-3′. PCR amplification was conducted in MyCycler (Bio-Rad). The reaction mixture was first subjected to 95°C for 3 minutes, followed by an optimal cycle of amplifications, consisting of 95°C for 30 seconds, optimal annealing temperature for 30 seconds, and 72°C for 30 seconds. Final extension was at 72°C for 10 minutes. PCR products were analyzed on 1.5% (w/v) agarose gels containing 0.5 µg/ml ethidium bromide.

### Measurement of pulmonary edema

As an index of lung edema, the amount of extravascular lung water was calculated according to established techniques [37], [38], [39]. Briefly, mice were killed 8 hours after surgery and blood was collected by cardiac puncture. The lungs were excised from mice, cleared of all extrapulmonary tissue, blotted and weighed (total lung wet weight); they were then dried in an incubator for 48 hours at 80°C and weighed again (total dry weight). For each animal, pulmonary edema was expressed as the ratio of total wet weight to total dry weight.

### Alanine aminotransferase (ALT) and aspartate aminotransferase (AST) assay

Plasma ALT and AST activities were measured with the Infinity ALT and AST liquid stable reagent (Thermo Electron Corporation, Pittsburgh, PA, USA) according to the manufacturer's instructions.

### Nuclear extraction and measurement of NF-κB activation

Nuclear extracts from lung (50 mg) and liver (100 mg) were prepared by using a nuclear extraction kit as described by the manufacturer (Active Motif, Carlsbad, CA, USA). Protein concentrations in nuclear extracts were determined using a Bradford assay (Bio-Rad). NF-κB activation was determined using TransAM NF-κB p65 transcription factor assay kit (Active Motif). The kit consists of a 96-well plate, into which oligonucleotide containing the NF-κB consensus site (5′-GGGACTTTCC-3′) is bound. The active form of NF-κB in the nuclear extract specifically binds to this consensus site and is recognized by a primary antibody specific for the activated form of p65 of NF-κB. An horseradish peroxidase-conjugated secondary antibody provides the basis for the colorimetric quantification. The absorbance of the resulting solution was measured 2 minutes later (450 nm with a reference wavelength of 655 nm), using a 96-well microplate reader (Tecan Systems, Mannedorf, Switzerland). The wild-type consensus oligonucleotide is provided as a competitor for NF-κB binding to monitor the specificity of the assay. Results were expressed as fold increase over the control group.

### Western immunoblot

Lung (50 mg) and liver (100 mg) tissues were homogenized at 4°C in 1 ml of radioimmunoprecipitation assay lysis buffer supplemented with 1% protease and 1% phosphatase inhibitor cocktail (Sigma-Aldrich), followed by centrifugation at 14,000 *g* for 10 minutes at 4°C. Protein concentration in the soluble fraction was determined by the Bradford method. Protein samples (50–100 µg) were separated by SDS-PAGE on Novex Bis-Tris polyacrylamide gels and transferred onto polyvinylidene difluoride membranes by electroblotting in Novex transfer buffer (Invitrogen Life Technologies) containing 20% (v/v) methanol. Membranes were then washed, blocked, and probed overnight at 4°C with rabbit anti-IκBα, phospho-IκBα, ERK_1/2_, and phospho-ERK_1/2_ (Cell Signaling Technology, Beverly, MA, USA; 1∶1000 dilution for all), followed by secondary detection for 2 hours with an horseradish peroxidase-conjugated, goat anti-rabbit IgG (Santa Cruz Biotechnology, CA, USA; 1∶1000 dilution). Membranes were washed and then incubated in SuperSignal West Pico chemiluminescent substrate (Pierce Biotechnology, Rockford, IL, USA) before exposure to X-ray films (LC-XPosure, Pierce Biotechnology). Gels were calibrated by protein kaleidoscope standards (Bio-Rad). Hypoxanthine-guanine phosphoribosyltransferase (HPRT) (Santa Cruz Biotechnology; 1∶2000 dilution) was applied as an internal control to normalize protein loading. The intensity of bands was quantified using LabWorks Image Analysis software (Ultra-Violet Products Ltd., Cambridge, UK).

### Statistics

The data were expressed as mean ± SEM. The significance of difference among groups was evaluated by one-way ANOVA with a post-hoc Tukey's test for multiple comparisons. A value of P<0.05 was regarded as statistically significant.

## Results

### Capsazepine attenuates endogenous SP concentrations in both septic and septic mice administrated with NaHS

**Figure 1:**
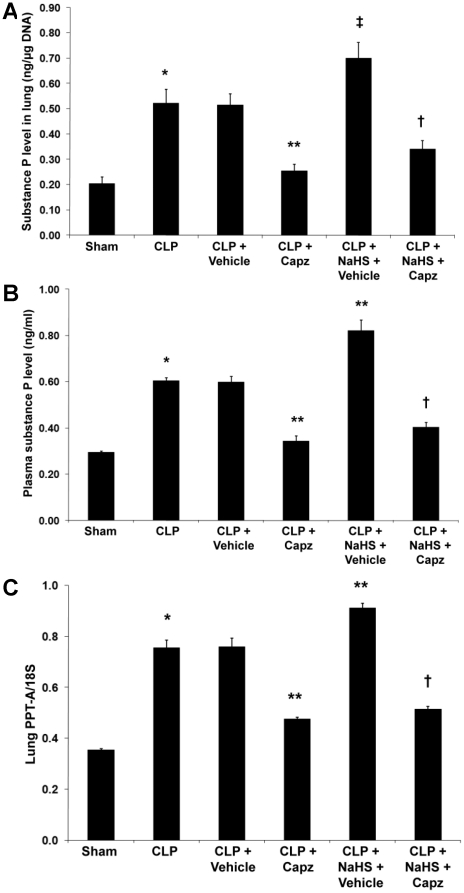
Effect of NaHS and capsazepine on protein and mRNA levels of SP in septic mice. Mice were randomly given NaHS (10 mg/kg, i.p.) or vehicle (DMSO) at the same time of CLP; and capsazepine (*Capz*) (15 mg/kg, s.c.) or vehicle (DMSO) 30 minutes before CLP. Sham mice were used as controls. Eight hours after CLP or sham operation, (A) lung and (B) plasma SP levels, and (C) lung PPT-A mRNA levels were measured. Results shown are the mean values ± SEM (n = 8–12 mice per group). *P<0.01 versus sham; **P<0.01 versus CLP+vehicle; ‡P<0.05 versus CLP+vehicle; †P<0.01 versus CLP+NaHS+vehicle.

The concentration of SP in both lung (Fig. 1A) and plasma (Fig. 1B) was significantly increased after induction of sepsis. Densitometric analysis of PCR products on agarose gel showed that pulmonary PPT-A mRNA expression correlated well with protein levels (Fig. 1C). Administration of capsazepine significantly suppressed lung (Fig. 1A) and plasma (Fig. 1B) SP levels. Consistently, transcriptional level of PPT-A gene expression was markedly reduced upon treatment with capsazepine (Fig. 1C). Administration of NaHS resulted in a further rise in the pulmonary (Fig. 1A) and plasma (Fig. 1B) SP levels in sepsis. Likewise, pulmonary mRNA level of PPT-A was significantly elevated in septic mice administrated with NaHS (Fig. 1C). Importantly, in the presence of NaHS, a significant attenuation of endogenous SP concentrations occurred in both lung (Fig. 1A) and plasma (Fig. 1B), consistent with a parallel decrease in pulmonary gene expression for SP (Fig. 1C) in septic mice treated with capsazepine.

### The attenuated SP levels correlates with reduced production of proinflammatory molecules in both septic and septic mice administrated with NaHS

**Figure 2:**
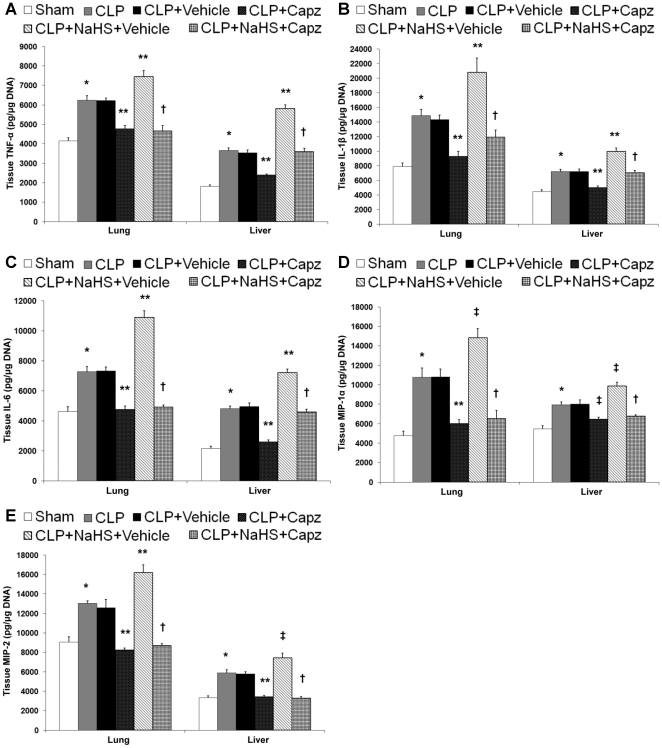
Effect of NaHS and capsazepine on protein levels of cytokines and chemokines in the lungs and liver of septic mice. Mice were randomly given NaHS (10 mg/kg, i.p.) or vehicle (DMSO) at the same time of CLP; and capsazepine (*Capz*) (15 mg/kg, s.c.) or vehicle (DMSO) 30 minutes before CLP. Sham mice were used as controls. Eight hours after CLP or sham operation, protein levels of (A) TNF-α, (B) IL-1β, (C) IL-6, (D) MIP-1α and (E) MIP-2 were measured. Results shown are the mean values ± SEM (n = 8–12 mice per group). *P<0.01 versus sham; **P<0.01 versus CLP+vehicle; ‡P<0.05 versus CLP+vehicle; †P<0.01 versus CLP+NaHS+vehicle.

**Figure 3:**
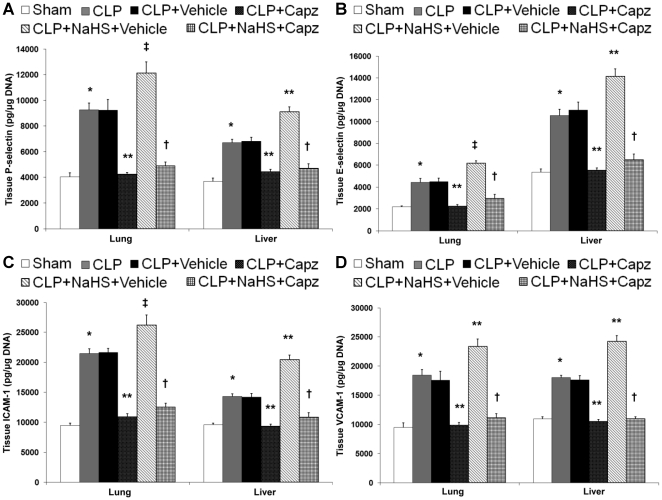
Effect of NaHS and capsazepine on protein levels of adhesion molecules in the lungs and liver of septic mice. Mice were randomly given NaHS (10 mg/kg, i.p.) or vehicle (DMSO) at the same time of CLP; and capsazepine (*Capz*) (15 mg/kg, s.c.) or vehicle (DMSO) 30 minutes before CLP. Sham mice were used as controls. Eight hours after CLP or sham operation, protein levels of (A) P-selectin, (B) E-selectin, (C) ICAM-1, and (D) VCAM-1 were measured. Results shown are the mean values ± SEM (n = 8–12 mice per group). *P<0.01 versus sham; **P<0.01 versus CLP+vehicle; ‡P<0.05 versus CLP+vehicle; †P<0.01 versus CLP+NaHS+vehicle.

The protein levels of proinflammatory cytokines, TNF-α, IL-1β, IL-6; chemokines, MIP-1α, MIP-2 (Fig. 2A–E); and adhesion molecules, P-selectin, E-selectin, ICAM-1, and VCAM-1 (Fig. 3A–D) in both lung and liver homogenates showed a marked rise in septic mice as compared with sham mice. All of these were significantly lowered by capsazepine (Fig. 2 and 3). Administration of NaHS in septic mice further enhanced the production of these mediators (Fig. 2 and 3) but capsazepine alleviated them (Fig. 2 and 3). At the transcriptional level, similar trends were observed that correlated well with the protein levels in both septic and septic mice administrated with NaHS (data not shown).

### Capsazepine protects against MODS in both septic and septic mice administrated with NaHS

**Figure 4:**
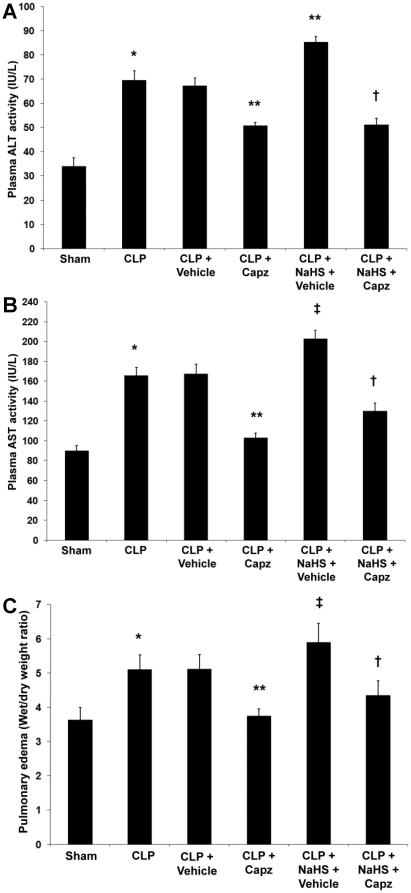
Effect of NaHS and capsazepine on liver dysfunction and lung edema in septic mice. Mice were randomly given NaHS (10 mg/kg, i.p.) or vehicle (DMSO) at the same time of CLP; and capsazepine (*Capz*) (15 mg/kg, s.c.) or vehicle (DMSO) 30 minutes before CLP. Sham mice were used as controls. Eight hours after CLP or sham operation, plasma levels of (A) ALT and (B) AST, and (C) lung wet-to-dry weight ratio were measured. Results shown are the mean values ± SEM (n = 10–15 mice per group). *P<0.01 versus sham; **P<0.01 versus CLP+vehicle; ‡P<0.05 versus CLP+vehicle; †P<0.01 versus CLP+NaHS+vehicle.

MODS is recognized to be the ultimate cause of death in patients with sepsis [11]. In an attempt to determine if TRPV1 antagonism by capsazepine is beneficial in preventing MODS, we determined pulmonary edema levels to assess the severity of lung injury [35], and measured plasma ALT and AST activities as indices of hepatic dysfunction [40]. Evidence of lung and liver damage after septic injury was confirmed by the heightened levels of pulmonary edema as indicated by wet-to-dry weight ratio (Fig. 4C), and increased activities of plasma ALT (Fig. 4A) and AST (Fig. 4B), respectively. However, septic mice treated with capsazepine showed significantly lower lung wet-to-dry weight ratio (Fig. 4C), and plasma ALT (Fig. 4A) and AST (Fig. 4B) activities. Administration of NaHS in septic mice further exacerbated these organ injury parameters (Fig. 4A–C) but capsazepine alleviated these deleterious effects (Fig. 4A–C).

### Capsazepine has no effect on PAG-mediated attenuation of SP levels in sepsis

**Figure 5:**
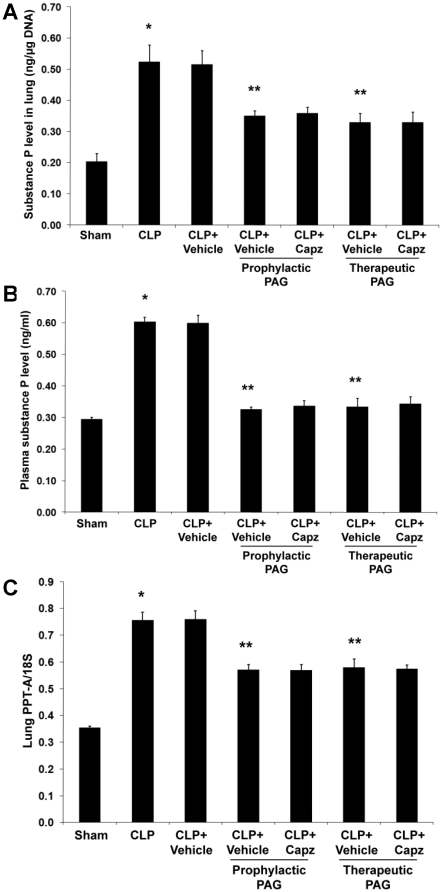
Effect of PAG and capsazepine on protein and mRNA levels of SP in septic mice. Mice were randomly given PAG (50 mg/kg, i.p.) 1 hour before (“prophylactic”) or 1 hour after (“therapeutic”) CLP; and capsazepine (*Capz*) (15 mg/kg, s.c.) or vehicle (DMSO) 30 minutes before CLP. Sham mice were used as controls. Eight hours after CLP or sham operation, (A) lung and (B) plasma SP levels, and (C) lung PPT-A mRNA levels were measured. Results shown are the mean values ± SEM (n = 8–12 mice per group). *P<0.01 versus sham; **P<0.01 versus CLP+vehicle.

Our data showed that prophylactic or therapeutic administration of PAG mitigated both pulmonary (Fig. 5A) and plasma (Fig. 5B) SP levels in sepsis. This is consistent with a parallel decrease in pulmonary gene expression for SP (Fig. 5C) in septic mice received PAG intervention. However, there were no significant differences in both the protein and transcriptional levels of SP in septic mice treated with both PAG and capsazepine when compared to their vehicle control counterparts (Fig. 5A–C).

### Inhibition of H_2_S formation impaired proinflammatory molecules production after septic injury, but capsazepine has no effect on them

**Figure 6:**
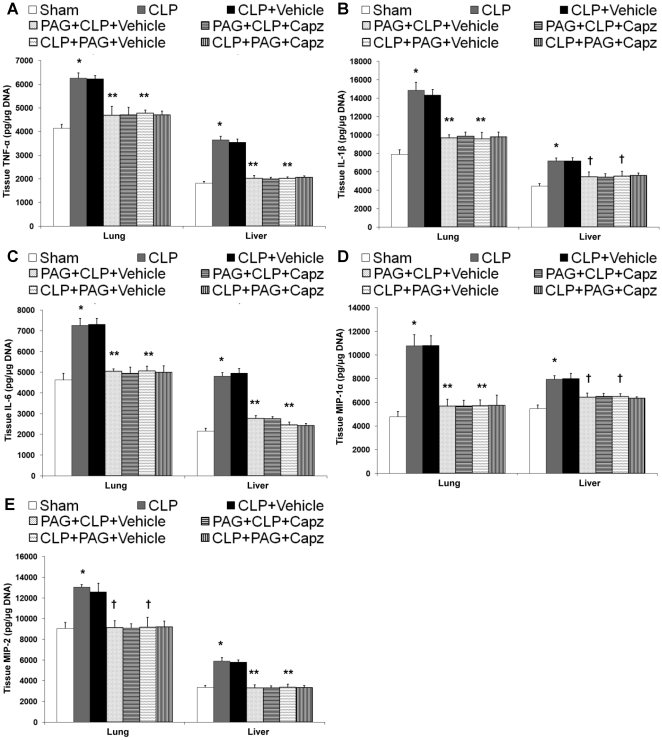
Effect of PAG and capsazepine on protein levels of cytokines and chemokines in the lungs and liver of septic mice. Mice were randomly given PAG (50 mg/kg, i.p.) 1 hour before (“prophylactic”; PAG+CLP+Vehicle or PAG+CLP+Capz) or 1 hour after (“therapeutic”; CLP+PAG+Vehicle or CLP+PAG+Capz) CLP; and capsazepine (*Capz*) (15 mg/kg, s.c.) or vehicle (DMSO) 30 minutes before CLP. Sham mice were used as controls. Eight hours after CLP or sham operation, protein levels of (A) TNF-α, (B) IL-1β, (C) IL-6, (D) MIP-1α and (E) MIP-2 were measured. Results shown are the mean values ± SEM (n = 8–12 mice per group). *P<0.01 versus sham; **P<0.01 versus CLP+vehicle; †P<0.05 versus CLP+vehicle.

**Figure 7:**
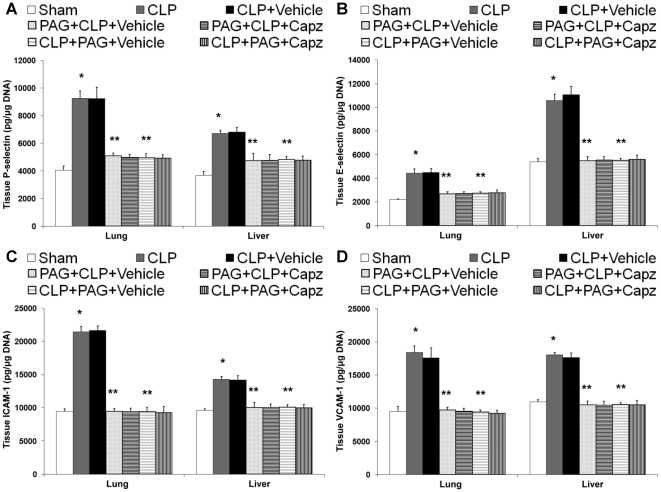
Effect of PAG and capsazepine on protein levels of adhesion molecules in the lungs and liver of septic mice. Mice were randomly given PAG (50 mg/kg, i.p.) 1 hour before (“prophylactic”; PAG+CLP+Vehicle or PAG+CLP+Capz) or 1 hour after (“therapeutic”; CLP+PAG+Vehicle or CLP+PAG+Capz) CLP; and capsazepine (*Capz*) (15 mg/kg, s.c.) or vehicle (DMSO) 30 minutes before CLP. Sham mice were used as controls. Eight hours after CLP or sham operation, protein levels of (A) P-selectin, (B) E-selectin, (C) ICAM-1, and (D) VCAM-1 were measured. Results shown are the mean values ± SEM (n = 8–12 mice per group). *P<0.01 versus sham; **P<0.01 versus CLP+vehicle.

We found that both pre- and post-treatment of PAG decreased protein levels of proinflammatory cytokines, TNF-α, IL-1β, IL-6; chemokines, MIP-1α, MIP-2 (Fig. 6A–E); and adhesion molecules, P-selectin, E-selectin, ICAM-1, and VCAM-1 (Fig. 7A–D) in both lung and liver homogenates, as compared with vehicle-injected septic mice that exhibited significant increments in the protein levels of these inflammatory mediators. A similar profile of the mRNA expression for these molecules was also observed (data not shown). However, there were no significant differences in the levels of these inflammatory mediators, at both the protein (Fig. 6 and 7) and transcriptional (data not shown) levels, in septic mice treated with both PAG and capsazepine when compared to those that received PAG only.

### Beneficial effects of capsazepine and PAG are not additive in protection against MODS in sepsis

**Figure 8:**
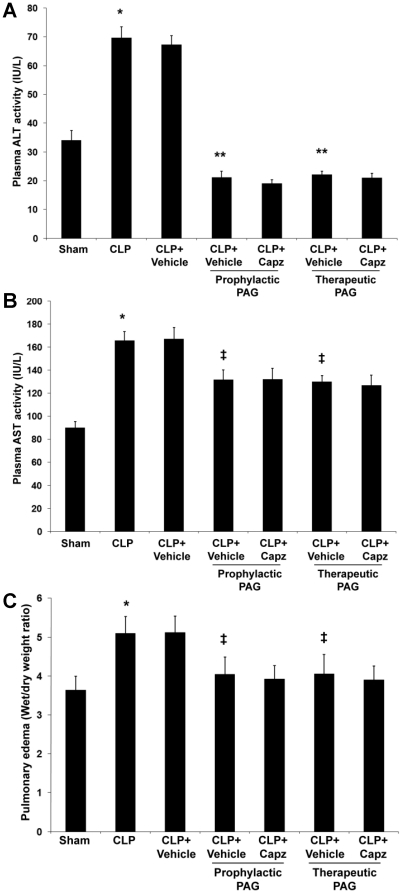
Effect of PAG and capsazepine on liver dysfunction and lung edema in septic mice. Mice were randomly given PAG (50 mg/kg, i.p.) 1 hour before (“prophylactic”) or 1 hour after (“therapeutic”) CLP; and capsazepine (*Capz*) (15 mg/kg, s.c.) or vehicle (DMSO) 30 minutes before CLP. Sham mice were used as controls. Eight hours after CLP or sham operation, plasma levels of (A) ALT and (B) AST, and (C) lung wet-to-dry weight ratio were measured. Results shown are the mean values ± SEM (n = 10–15 mice per group). *P<0.01 versus sham; **P<0.01 versus CLP+vehicle; ‡P<0.05 versus CLP+vehicle.

Further evidence of neurogenic contribution of H_2_S in mediating sepsis-associated MODS was demonstrated by non-significant changes of pulmonary edema levels, plasma ALT and AST activities in septic mice treated with both PAG and capsazepine, as compared with their vehicle control counterparts (Fig. 8A–C). These data demonstrated that absence of H_2_S alone is sufficient to ameliorate lung and liver dysfunction as indicated by decreased levels of lung wet-to-dry weight ratio (Fig. 8C), and reduced activities of plasma ALT (Fig. 8A) and AST (Fig. 8B), respectively.

### Effect of capsazepine on ERK_1/2_ activation in H_2_S-induced neurogenic inflammation in sepsis

**Figure 9:**
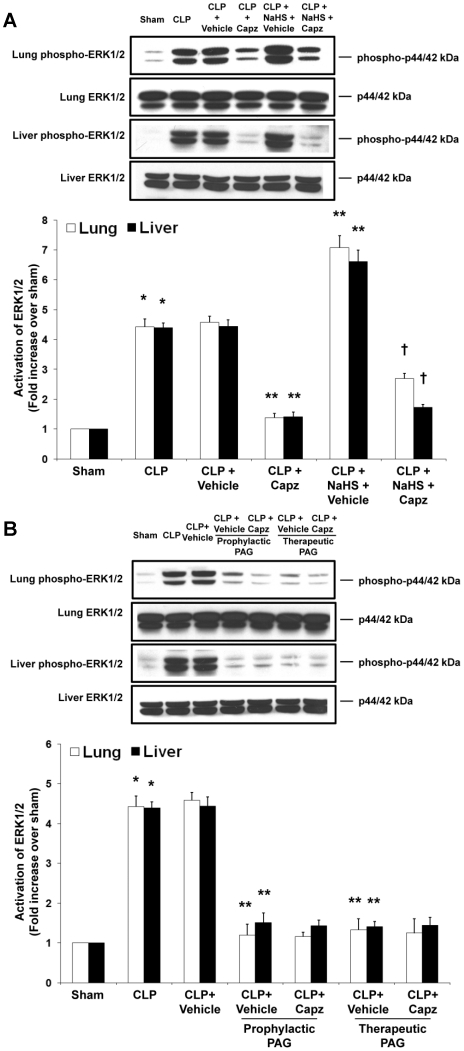
Effect of NaHS or PAG and capsazepine on ERK_1/2_ activation in the lungs and liver of septic mice. Mice were randomly given NaHS (10 mg/kg, i.p.) at the same time of CLP or PAG (50 mg/kg, i.p.) 1 hour before (“prophylactic”) or 1 hour after (“therapeutic”) CLP; and capsazepine (*Capz*) (15 mg/kg, s.c.) or vehicle (DMSO) 30 minutes before CLP. Sham mice were used as controls. Eight hours after CLP or sham operation, effect of (A) NaHS or (B) PAG and capsazepine on phospho-ERK_1/2_ and total ERK_1/2_ expression levels were measured. A representative western blot image is shown, with densitometry data expressed as average ratios of phospho-ERK_1/2_ to total ERK_1/2_. Results shown are the mean values ± SEM (n = 6 mice per group). *P<0.01 versus sham; **P<0.01 versus CLP+vehicle; †P<0.01 versus CLP+NaHS+vehicle.

To investigate the signaling mechanisms by which H_2_S regulates TRPV1-mediated neurogenic inflammation in sepsis, we evaluated the ERK_1/2_ and NF-κB signaling pathways, with the usage of capsazepine, using two different and complementary approaches: exogenous administration of NaHS as an H_2_S donor and inhibition of endogenous H_2_S formation by PAG. We observed activation of pulmonary and hepatic ERK_1/2_ in septic mice as compared with sham mice (Fig. 9A). Upon treatment of capsazepine, phosphorylation of ERK_1/2_ in lung and liver were abolished (Fig. 9A). Administration of exogenous NaHS further enhanced tissue ERK_1/2_ activation in sepsis, whereby capsazepine treated, NaHS-injected septic mice revealed significantly reduced lung and liver phospho-ERK_1/2_ expressions (Fig. 9A). Additionally, prophylactic or therapeutic administration of PAG markedly suppressed tissue phosphorylation of ERK_1/2_ in sepsis (Fig. 9B). Interestingly, the status of ERK_1/2_ activation in septic mice administrated with both PAG and capsazepine remained unchanged when compared to those that received PAG only (Fig. 9B).

### Effect of capsazepine on IκBα phosphorylation and degradation levels and NF-κB activity in H_2_S-induced neurogenic inflammation in sepsis

**Figure 10:**
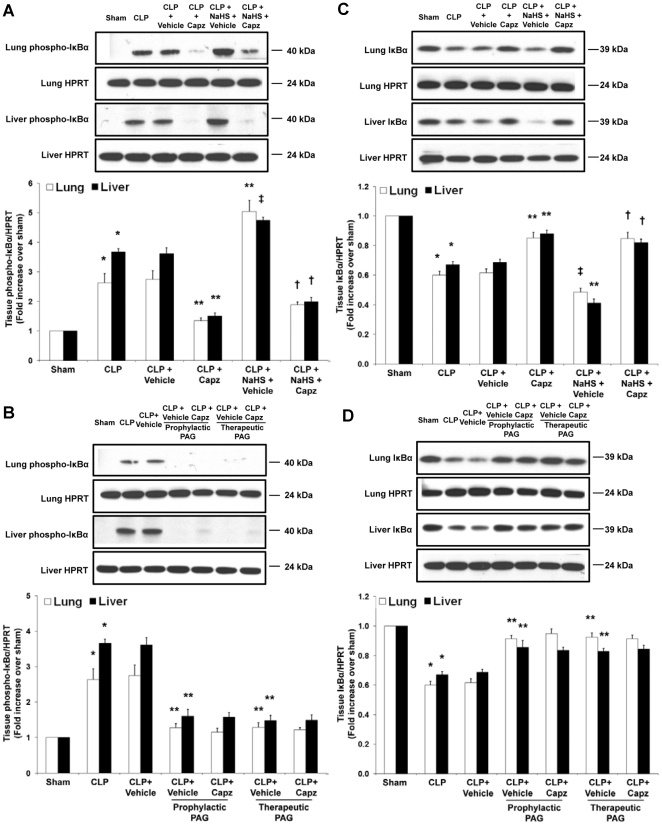
Effect of NaHS or PAG and capsazepine on IκBα phosphorylation and degradation levels in the lungs and liver of septic mice. Mice were randomly given NaHS (10 mg/kg, i.p.) at the same time of CLP or PAG (50 mg/kg, i.p.) 1 hour before (“prophylactic”) or 1 hour after (“therapeutic”) CLP; and capsazepine (*Capz*) (15 mg/kg, s.c.) or vehicle (DMSO) 30 minutes before CLP. Sham mice were used as controls. Eight hours after CLP or sham operation, effect of NaHS or PAG and capsazepine on (A and B) phospho-IκBα and (C and D) IκBα expression levels were measured. A representative western blot image is shown, with densitometry data expressed as average ratios of phospho-IκBα to HPRT and IκBα to HPRT levels. Results shown are the mean values ± SEM (n = 6 mice per group). *P<0.01 versus sham; **P<0.01 versus CLP+vehicle; ‡P<0.05 versus CLP+vehicle; †P<0.01 versus CLP+NaHS+vehicle.

Subsequently, we analyzed whether ERK_1/2_ signaling leads to activation of inhibitory κBα (IκBα) and NF-κB in H_2_S-induced neurogenic inflammation in sepsis. Capsazepine greatly reduced pulmonary and hepatic levels of phospho-IκBα in septic mice as compared to the elevated levels detected in untreated septic mice (Fig. 10A). Consistently, phospho-IκBα levels in the lungs and liver of septic mice injected with NaHS were greatly enhanced; while these levels were significantly suppressed by capsazepine (Fig. 10A). In contrast, PAG intervention drastically reduced lung and liver phospho-IκBα levels in sepsis and capsazepine has no effect on them (Fig. 10B).

More importantly, assessment of IκBα degradation displayed a similar profile that correlated well with the levels of phospho-IκBα, with untreated septic mice showing a significant reduction in lung and liver IκBα levels in comparison with the heightened levels exhibited in capsazepine treated septic mice (Fig. 10C). Likewise, the expression levels of IκBα in lung and liver tissues from septic mice injected with NaHS were again lowered; while these levels were elevated with capsazepine (Fig. 10C). Additionally, treatment with PAG markedly enhanced tissue IκBα expression levels in comparison to their vehicle control counterparts, suggesting an obvious inhibition of IκBα degradation (Fig. 10D). However, treatment of capsazepine failed to further modulate these levels (Fig. 10D).

**Figure 11:**
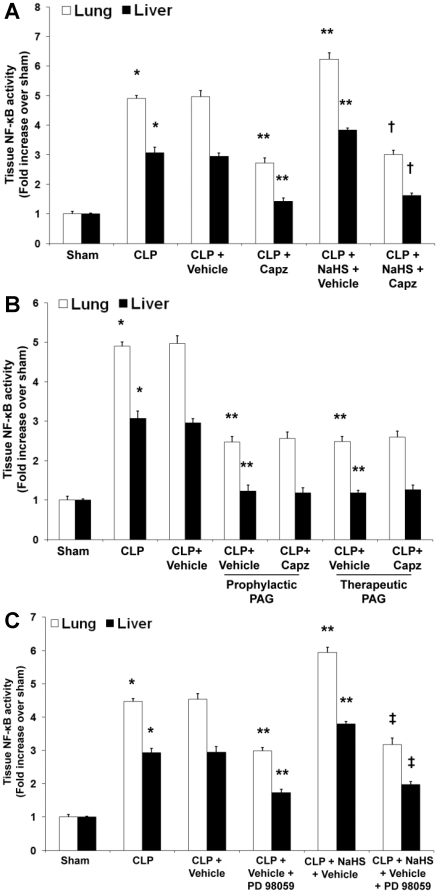
Effect of NaHS or PAG and capsazepine and effect of NaHS and PD98059 on NF-κB activation in nuclear extracts of lung and liver tissues in septic mice. Mice were randomly given NaHS (10 mg/kg, i.p.) at the same time of CLP or PAG (50 mg/kg, i.p.) 1 hour before (“prophylactic”) or 1 hour after (“therapeutic”) CLP; and capsazepine (*Capz*) (15 mg/kg, s.c.) or vehicle (DMSO) 30 minutes before CLP. Some mice were injected PD98059 (10 mg/kg, i.p.) or vehicle (DMSO) 1 hour before CLP. Sham mice were used as controls. Eight hours after CLP or sham operation, effect of (A) NaHS or (B) PAG and capsazepine and (C) effect of NaHS and PD98059 on the DNA-binding activity of NF-κB were measured. Results shown are the mean values ± SEM (n = 8–12 mice per group). *P<0.01 versus sham; **P<0.01 versus CLP+vehicle; †P<0.01 versus CLP+NaHS+vehicle. ‡P<0.01 versus CLP+NaHS+vehicle.

Next, we examined whether phosphorylation and degradation of IκBα result in NF-κB nuclear translocation. Our results showed that capsazepine significantly decreased the DNA-binding activity of nuclear NF-κB in the lungs and liver of septic mice (Fig. 11A). Exogenous administration of NaHS further amplified the activation of NF-κB while treatment with capsazepine significantly disrupted the activity of NF-κB (Fig. 11A). In the absence of endogenous H_2_S, the DNA-binding activity of nuclear NF-κB was greatly reduced (Fig. 11B). As anticipated, we noticed no difference in lung and liver NF-κB activation from septic mice treated with both PAG and capsazepine as compared with their vehicle control counterparts (Fig. 11B).

Finally, to examine if ERK_1/2_ activation occurs upstream of NF-κB, we measured tissue NF-κB activity in the presence of PD98059, a potent and selective antagonist of MEK-1 that is the upstream kinase of ERK_1/2_ [28], [29]. Our results showed that PD98059 significantly decreased the DNA-binding activity of nuclear NF-κB in the lungs and liver of septic mice (Fig. 11C). In the presence of NaHS, a marked attenuation of tissue NF-κB activation by PD98059 was observed in sepsis, thus providing convincing evidence that ERK_1/2_ activation occurs upstream of NF-κB (Fig. 11C).

## Discussion

Sepsis remains an important global healthcare problem even in the modern era of critical care management. Thus, identifying endogenous neural elements that modulate H_2_S-induced neurogenic inflammation in sepsis and elucidating the underlying molecular mechanisms by which this occurs are of paramount importance. Our previous study demonstrated that H_2_S promotes TRPV1-mediated neurogenic inflammation in polymicrobial sepsis [10]. In the present study, we showed that overproduction of SP significantly increases the severity of sepsis in a TRPV1-dependent manner; and that this phenomenon occurred under the influences of the proinflammatory effects of H_2_S. Additionally, we found that the underlying signal transduction pathway by which H_2_S, SP and TRPV1 interact to instigate neural inflammatory processes involve activation of the ERK-NF-κB pathway.

In recent years, the role of SP in the regulation of inflammatory conditions in lipopolysaccharide-evoked endotoxemia, CLP-induced sepsis, as well as human sepsis and septic shock has been suggested [41], [42], [43]. Of even greater significance, H_2_S has been shown to implicate in neurogenic inflammation mediated by SP and TRPV1. By stimulating TRPV1-expressing sensory neurons, H_2_S provoked the release of SP from isolated guinea pig airways [5]. Likewise, heightened circulatory level of SP was observed in normal mice administrated with NaHS [19]. Notably, in both studies, SP levels were greatly attenuated by capsaicin desensitization or by capsazepine administration. Besides, H_2_S induced contraction in rat urinary bladder *via* a neurogenic mechanism that involved TRPV1 stimulation with consequent release of tachykinin [44], [45]. Furthermore, endogenous H_2_S was found to modulate sepsis-associated lung injury and caerulein-induced acute pancreatitis through upregulation of SP in the lungs and pancreas, respectively [43], [46]. Here, we show that H_2_S regulates TRPV1-mediated neurogenic inflammation in sepsis through upregulation of pulmonary and plasma SP. Our findings are consistent with earlier observations and reinforce the essential role of SP, and its interaction with H_2_S. It must be noted, however, that with the usage of capsazepine, a synthetic competitive antagonist of TRPV1; our results provide the first pharmacological evidence that H_2_S provokes tachykinin-mediated neurogenic inflammatory responses involving SP in sepsis in a TRPV1-dependent manner.

In the first report showing the relations between H_2_S and infection, endogenous vascular H_2_S content was increased in rats with septic and endotoxic shock. It was suggested that excess H_2_S production was involved in pathophysiological processes during shock [9]. Consistently, our previous study has shown that CLP-induced sepsis significantly increased the plasma H_2_S level and the liver CSE activity in septic mice. Prophylactic and therapeutic administration of PAG in septic mice completely abolished the CSE synthesizing activity in the liver, thereby leading to a significant reduction in plasma H_2_S level [10]. Furthermore, capsazepine has no effect on endogenous generation of H_2_S in sepsis. Employing the same experimental approach, it was demonstrated that hepatic CSE activity and plasma H_2_S level in septic mice treated with capsazepine and those treated with both PAG and capsazepine were comparable to their vehicle control counterparts [10]. Here, we report that capsazepine significantly attenuated endogenous SP levels in both septic and septic mice administrated with NaHS. Moreover, capsazepine has no effect on PAG-mediated attenuation of SP level in sepsis. Taken together, these findings suggest that endogenous H_2_S signaled *via* TRPV1 which resulted in the subsequent release of SP, thereby allowing SP to elicit its potent proinflammatory effects in sepsis. It is worth mentioning that TRPV1 is broadly expressed in all “port of entry” tissues such as the skin, gut, airway and conjunctiva [2], [47], hence we restricted measurement of SP to the lungs. Nevertheless, a systemic role of SP *via* the circulatory system was investigated in this study to substantiate its generalized role in mediating neurogenic inflammation in sepsis.

SIRS and MODS are major hallmarks of sepsis. Excessive synthesis of inflammatory molecules in organs remote from the initial insult synergistically interact to mediate tissue damage, followed by cardiovascular collapse, MODS, and death of the host [11]. The current study revealed that pharmacological blockade of TRPV1 markedly reduced pulmonary and hepatic levels of IL-1β, IL-6, TNF-α, MIP-1α, MIP-2, P-selectin, E-selectin, VCAM-1 and ICAM-1 in both septic and septic mice administrated with NaHS. Likewise, a significant protection against lung injury and liver dysfunction, as indicated by pulmonary edema and serum ALT and AST activities respectively, was conferred by capsazepine. Importantly, both observations corresponded with concurrent attenuation of SP level, suggesting that H_2_S stimulation of TRPV1 and the subsequent release of SP can induce upregulation of proinflammatory mediators that implicated in SIRS and work in conjunction with them to initiate MODS. The observed protection against MODS was further associated with improved survival benefits, as capsazepine has been previously reported to reduce mortality in sepsis even in the presence of NaHS [10]. To this regard, it should be noted that lung and liver were chosen for investigation primarily because lungs are often the first organ to succumb post systemic insults, and liver represents an important organ not only in host defense [48], but in addition that CSE is highly expressed in hepatocytes [49]. Collectively, our results established H_2_S stimulation of TRPV1 and the downstream release of SP as a key element in the transition of infection and SIRS to MODS in sepsis, and identified TRPV1 antagonist as a possible therapeutic target for the treatment of polymicrobial sepsis.

In contrast, beneficial effects of TRPV1 antagonism in ameliorating SIRS and MODS in sepsis were diminished when endogenous synthesis of H_2_S was blocked as seen in septic mice received PAG intervention as compared to the same mice treated with capsazepine. It appears that inhibition of H_2_S generation alone is sufficient to account for these observations, and that the presence or absence of TRPV1 antagonism makes no difference. Nonetheless, with regard to the inhibitory effects of CSE synthesizing activity, it should be underlined that the effects of PAG alone seem not to be a consequence of blood pressure changes, given that at the doses used PAG did not significantly change blood pressure [50]. Besides, the effect of capsazepine is not entirely secondary to an enhanced formation of H_2_S. Since sepsis is a multifactorial disease, other inflammatory mediators may stimulate TRPV1 and trigger the release of SP. Nevertheless, we ascertained from our data that sepsis has a significant sensory neurogenic component that involved SP and mediated by H_2_S in a TRPV1-dependent manner.

Although convincing data have suggested the relevance of ERK_1/2_ signaling to H_2_S, their association with TRPV1 remains unknown [18], [51], [52], [53]. In the current study, we showed that pharmacological antagonism of TRPV1 inhibits phosphorylation of ERK_1/2_ and IκBα, concurrent with inhibition of IκBα degradation and DNA binding activity of nuclear NF-κB, in both septic and septic mice administrated with NaHS. Conversely, in the absence of H_2_S, capsazepine has no effect on PAG-mediated suppression of these parameters in sepsis. These findings not only indicate that H_2_S, SP and TRPV1 interact to instigate neurogenic inflammation in sepsis through activation of the ERK-NF-κB pathway, but also added to the growing evidence about the involvement of ERK_1/2_ in regulating the activity of NF-κB [14], [15], and the potential association between H_2_S and ERK-NF-κB [18], [51], [52], [53]. Furthermore, the observed suppression of tissue NF-κB activation in the presence of PD98059, a selective inhibitor of MEK-1 that is the upstream kinase of ERK_1/2_, directly ascertained that ERK_1/2_ signaling occurs upstream of NF-κB activation. Reinforcing our findings that H_2_S induces TRPV1-mediated neurogenic inflammation through activation of the ERK-NF-κB pathway, H_2_S has been reported previously, using specific inhibitors for ERK and NF-κB, to alleviate tissue inflammatory cytokines and chemokines production in septic mice and normal mice injected with NaHS, respectively [18], [54]. Nevertheless, H_2_S has been linked to a number of other pathways [55], [56], [57], [58], hence, alternative mechanisms may exist which act in synergism with ERK signaling cascade to modulate the activity of NF-κB in sepsis. Additionally, since polyclonal antibody against p65 subunit was employed to investigate the activity of NF-κB, our results demonstrate that NF-κB dimers containing p65 may play a crucial role in H_2_S-provoked inflammation and injury response seen in this study.

Our findings suggest a proinflammatory role of H_2_S in sepsis, however, a recent paper by Spiller *et al.* [59] reports the anti-inflammatory properties of H_2_S. The inconsistency may be a result of the dose of NaHS used. We used NaHS at a dose of 10 mg/kg, which increased plasma concentration of H_2_S significantly and caused obvious lung and liver inflammation [10], [19], [26] whereas study by Spiller *et al.* used three doses of NaHS (10, 30, or 100 µmol/kg) that approximated the physiological concentrations of H_2_S. Importantly, it has been suggested that low (physiological) concentrations of H_2_S tend to be cytoprotective and anti-inflammatory while higher concentrations are likely to be cytotoxic and proinflammatory [6]. In addition, experimental differences that include health status, age, and gender of the animals; commercial sources of NaHS and PAG; and level of severity of sepsis may explain the opposing findings between us and them.

Although the present study offers the possibility that H_2_S may activate TRPV1, the precise molecular site of action and the molecular mechanisms underlying it remain unknown. It could be possible that the neuropeptide release observed with H_2_S is *via* an indirect activation of TRPV1 through other endogenous mediators evoked by H_2_S. Furthermore, the specific cell types that respond to H_2_S stimulation of TRPV1 activation, as well as the types of cells that bind to SP and mediate ERK-NF-κB signaling, remain to be identified. Molecular mechanisms that lead to reduced ERK_1/2_ phosphorylation and NF-κB activation with PAG are issues deserving further studies. Finally, investigation on the role of H_2_S in pathological conditions has been hampered by the paucity of specific pharmacological tools. Most irreversible inhibitors of CSE, including PAG, are of low potency, of low selectivity and of limited cell-membrane permeability that also non-specifically interfere with other pyridoxal-5′-phosphate-dependent enzymes [6], [8]. It is clear that newer and more promising chemical tools are required to probe deeper the complex biological roles of H_2_S.

**Figure 12:**
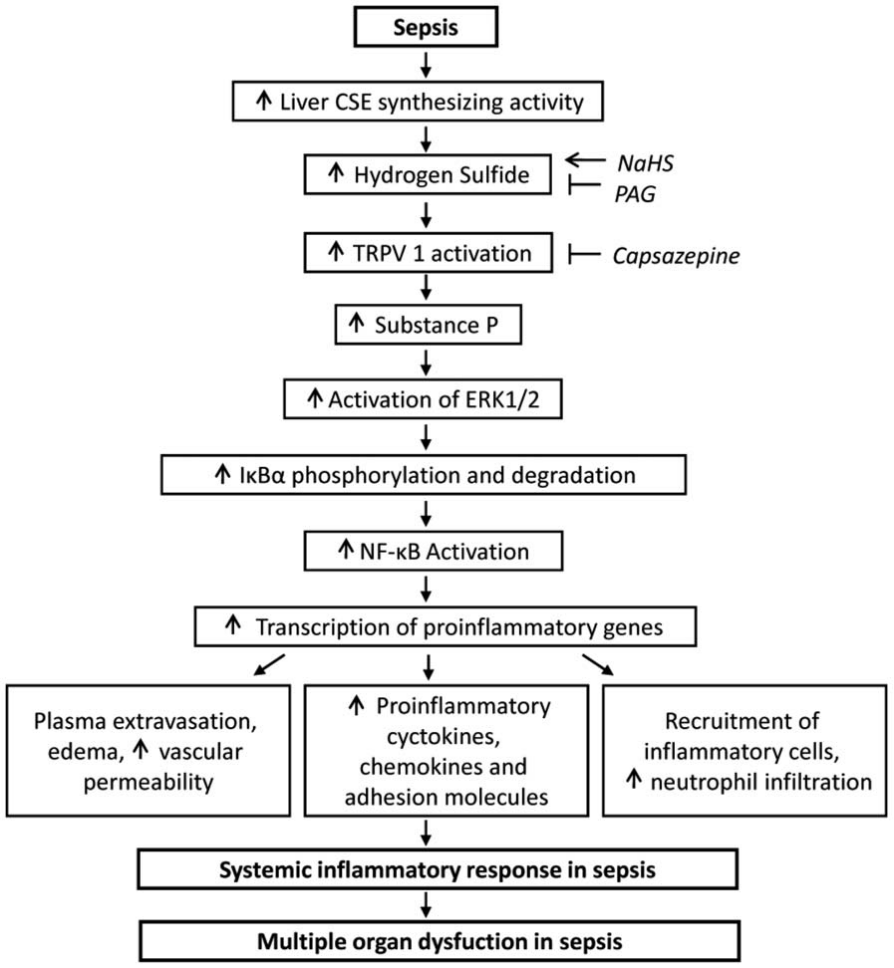
Schematic summary of signaling events in H_2_S-induced neurogenic inflammation in a murine model of polymicrobial sepsis. H_2_S has been demonstrated to be overproduced in sepsis. H_2_S stimulation of TRPV1 and the downstream release of SP lead to the activation of ERK_1/2_, which subsequently induces the phosphorylation and degradation of IκBα, as well as the translocation and activation of NF-κB, thereby leading to SIRS and MODS characteristic of severe sepsis. 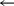 indicates exogenous administration; 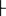 indicates inhibition.

In conclusion, we propose that H_2_S regulates TRPV1-mediated neurogenic inflammation in polymicrobial sepsis through enhancement of SP production and activation of the ERK-NF-κB pathway. Importantly, the H_2_S-TRPV1-SP-ERK_1/2_-IκBα-NF-κB signal transduction pathway contributes to SIRS and MODS in sepsis (Fig. 12). Collectively, our results contribute to a better understanding of the precise mechanism underlying the proinflammatory effects of H_2_S in the pathophysiology of sepsis in a TRPV1 relevance context and provide further insight into the development of new therapeutic intervention for sepsis and other inflammatory pathologies.
